# Binge Eating and Weight-Related Quality of Life in Obese Adolescents

**DOI:** 10.3390/nu4030167

**Published:** 2012-03-06

**Authors:** Lisa M. Ranzenhofer, Kelli M. Columbo, Marian Tanofsky-Kraff, Lauren B. Shomaker, Omni Cassidy, Brittany E. Matheson, Ronette L. Kolotkin, Jenna M. Checchi, Margaret Keil, Jennifer R. McDuffie, Jack A. Yanovski

**Affiliations:** 1 Section on Growth and Obesity, Program in Developmental Endocrinology and Genetics, *Eunice Kennedy Shriver* National Institute of Child Health and Human Development (NICHD), National Institutes of Health (NIH), DHHS, Bethesda, MD 20892, USA; Email: Lisa.Ranzenhofer@usuhs.mil (L.M.R.); k_columbo@yahoo.com (K.M.C.); shomakel@mail.nih.gov (L.B.S.); omni.cassidy@nih.gov (O.C.); brittany.matheson@nih.gov (B.E.M.); jenchec@gmail.com (J.M.C.); keilm@mail.nih.gov (M.K.); yanovskj@mail.nih.gov (J.A.Y.); 2 Uniformed Services University of the Health Sciences (USUHS), Bethesda, MD 20814, USA; 3 Department of Community and Family Medicine, Duke University Health Systems, Durham, NC 27710, USA; Email: rkolotkin@qualityoflifeconsulting.com (R.L.K.); mcduf.j@duke.edu (J.R.M.); 4 Obesity and Quality of Life Consulting, Durham, NC 27705, USA

**Keywords:** weight-related quality of life (WR-QOL), binge eating, adolescent, obesity

## Abstract

Limited data exist regarding the association between binge eating and quality of life (QOL) in obese adolescent girls and boys. We, therefore, studied binge eating and QOL in 158 obese (BMI ≥ 95th percentile) adolescents (14.5 ± 1.4 years, 68.0% female, 59% African-American) prior to weight-loss treatment. Youth completed an interview to assess binge eating and a questionnaire measure of QOL. Controlling for body composition, binge eating youth (*n* = 35), overall, reported poorer QOL in domains of health, mobility, and self-esteem compared to those without binge eating (*p*s < 0.05). Also, girls, overall, reported poorer QOL than boys in activities of daily-living, mobility, self-esteem, and social/interpersonal functioning (*p*s < 0.05). Girls with binge eating reported the greatest impairments in activities of daily living, mobility, self-esteem, social/interpersonal functioning, and work/school QOL (*p*s < 0.05). Among treatment-seeking obese adolescents, binge eating appears to be a marker of QOL impairment, especially among girls. Prospective and treatment designs are needed to explore the directional relationship between binge eating and QOL and their impact on weight outcomes.

## 1. Binge Eating and Weight-Related Quality of Life in Obese Adolescents

Binge eating is defined as consuming an objectively and contextually large amount of food, accompanied by the feeling of loss of control over eating [1]. Binge eating is the most common disordered eating behavior among obese adolescents, with rates often exceeding 35% among weight-loss treatment-seeking samples [2]. Among adolescents, binge eating has been associated with greater psychological and eating-related distress [2]. Despite these findings, and a consistent body of research indicating that obese youth experience poorer quality of life (QOL) than their non-overweight peers [3,4,5], there is a surprising dearth of research examining the relationship between binge eating and QOL among obese adolescents. 

Health-related QOL refers to the impact of an individual’s health on their QOL [6]. Data from adult studies suggest a unique association between binge eating and impairment in health-related QOL, independent of body weight [7,8,9]. For instance, obese adults with binge eating disorder report more impaired mental and physical QOL compared to obese adults without binge eating disorder [7,9]. To date, there are only two studies that have examined the relationship between disordered eating patterns and QOL in adolescents. In one study, overweight adolescents identified as being at high-risk for developing an eating disorder (including binge eating disorder) by virtue of elevated body weight and shape concerns reported inferior QOL in physical, emotional and social domains compared to adolescents categorized as low-risk [10]. In another recent study of youth with type 2 diabetes, those identified as binge eaters based upon responses to a questionnaire reported poorer QOL than youth without binge eating [11]. However, because adiposity was not accounted for among diabetic youth, it is unclear whether the link between binge eating and poorer QOL resulted from greater adiposity among those with binge eating. A consistent body of research indicates that obese youth experience poorer QOL than their non-overweight peers [3,4,5].

Given the high prevalence of obesity within the general population [12] and the substantial rates of binge eating among overweight youth [2], we examined the association between binge eating and weight-related QOL in adolescents seeking weight-loss treatment. Weight-related QOL, referring to the specific impact of body weight on a person’s QOL, is a component of health-related QOL particularly relevant for individuals with obesity [13]. Weight-related QOL is comprised of at least seven different domains of functioning including: physical “health” (e.g., “I am worried about my health”), “social/interpersonal functioning” with family and friends (e.g., “I have difficulty making friends”), “work/school” or academics (e.g., “I do not enjoy my school/work”), “mobility” or physically moving (e.g., “I feel clumsy or awkward”), “self-esteem” or feelings about oneself (e.g., “I am self-conscious”), “activities of daily living” or day-to-day tasks such as dressing (e.g., “I have difficulty finding clothes to fit me”), and “comfort with food” or relationship with food (e.g., “Food is a source of comfort and gratification”). Data suggest that adolescents who report binge eating have elevated disordered eating attitudes and cognitions, depressive symptoms, poorer family and social functioning, and poorer global self-esteem and perceptions of physical appearance, compared to those without binge eating [14,15,16,17,18]. There are also data that obesity is associated with these same variables [4,5,19,20]. We therefore hypothesized that obese adolescents with binge eating would report poorer weight-related QOL in the following domains of HR-QOL: Social/interpersonal functioning, self-esteem, work/school, activities of daily living, and comfort with food, than those without binge eating. Given numerous physical health consequences of obesity, including medical comorbidies, sleep problems and joint problems [21], we hypothesized that obese youth would have poorer QOL in domains of mobility and health.

Additionally, we investigated sex-related differences in adolescents’ QOL. We focused upon binge eating as objectively large episodes because they appear to be the most salient among obese, treatment-seeking youth [22]. Since depressive symptoms are strongly correlated with both obesity and binge eating [15,16,23,24,25], we also analyzed the potential contribution of depression in our examination of binge eating and QOL. Because some work suggests the salience of loss of control (LOC) independent of episode size, we also analyzed the association of LOC (objective or subjective binge eating episodes) with QOL in secondary analyses. 

## 2. Methods and Procedures

### 2.1. Participants

Participants were obese (≥95th BMI kg/m^2^ percentile) [26] African American and White, non-Hispanic boys and girls studied prior to participation in a weight loss study. All participants were recruited through newspaper advertisements and letters to physicians practicing within 60 miles of Bethesda, MD advertising a weight loss study involving medication. Youth qualified for participation if they were between the ages of 12–17 years, were classified as obese and had at least one quantifiable obesity-related comorbidity: hypertension, type 2 diabetes or impaired glucose tolerance, hyperinsulinemia, hyperlipidemia, hepatic steatosis, or sleep apnea. Individuals were excluded if they had a major pulmonary, hepatic, cardiac, or musculoskeletal disorder unrelated to obesity, history of substance abuse or other psychiatric disorder that would impair compliance with the study protocol, used an anorexiant in the past 6 months, or recently lost ≥5% of body weight. For a complete description of study requirements, see McDuffie *et al.*, 2004 [27]. This protocol was approved by the *Eunice Kennedy Shriver* National Institute of Child Health and Human Development Institutional Review Board. QOL measures from 110 participants have been reported previously in a study examining racial differences in QOL among overweight youth [28]. 

### 2.2. Procedure

All data were collected at baseline prior to the initiation of any treatment. Binge eating was assessed with the *Eating Disorder Examination version 12OD/C.2* (*EDE*) [29]. The EDE is a semi-structured clinical interview used to assess disordered attitudes and behaviors related to eating, body shape and weight, and specific DSM-IV-TR eating disorders [1]. The EDE has been successfully administered to adolescent samples [22]. The child version of the EDE differs from the adult EDE only in that its language is slightly altered in order to make it more accessible to children, and that two items assessing critical over-value of shape and body weight have been supplemented with a sorting task. Despite minor differences, the EDE and Child EDE capture the same constructs and have been successfully combined in prior studies [30,31]. In the current study, all participants age 13 and older were administered an adult EDE. If a participant had difficulty understanding a question, language from the Child EDE was substituted (even if the participant was over 13 years old). Our team has successfully used this approach to ensure that all participants understand the questions [30,31]. Both versions of the EDE were administered by trained graduate clinical psychology students and post-undergraduate research associates who attended 15–20 h of training. Before conducting interviews, each interviewer was trained by listening to audiotapes of sample interviews, conducting a practice interview, observing a trainer conducting an EDE, and then conducting an EDE while a trainer observed.

The EDE overeating section was used to identify the presence (*vs.* absence) of three types of eating episodes: Objective binge episodes (overeating accompanied by the experience of loss of control; LOC), subjective binge episodes (episodes of LOC but the amount of food eaten is not considered unambiguously large), and objective overeating episodes (overeating occurring in the absence of LOC). Participants were classified as having presence or absence of binge eating based upon whether they reported at least one objective binge episode in the 28 days prior to assessment. Youth with subjective binge eating, objective overeating, or episodes involving neither overeating nor LOC were grouped together. In secondary analyses, youth with objective and subjective binge episodes were grouped together into a “LOC” group, while youth with objective overeating or episodes involving neither overeating nor LOC were grouped as “no LOC”. The EDE has excellent psychometric properties [22]. Inter-rater reliability from a subset of participants ranged from 0.87 to 0.98 on the EDE subscale and total scores (*p*s < 0.01) [22]. 

*Impact of Weight on Quality of Life, Adapted for Use with Adolescents (IWQOL-A)* is a 66-item self-report instrument that measures seven domains of QOL: Health (e.g., “I am worried about my health”), social/interpersonal functioning (e.g., “I have difficulty making friends”), work/school (e.g., “I do not enjoy my schoolwork”), mobility (e.g., “I feel clumsy or awkward”), self-esteem (e.g., “I am self-conscious”), activities of daily living (e.g., “I have difficulty finding clothes to fit me”), and comfort with food (e.g., “Food is a source of comfort and gratification”). Items are rated on a 5-point Likert scale, and scales are derived by summing items. The possible subscale score ranges are as follows, with higher scores indicating greater impairment: Health, 12 to 60; social/interpersonal functioning, 11 to 55; work/school, 6 to 30; mobility, 10 to 50; self-esteem, 10 to 50; and activities of daily living, 7 to 35. For comparison, treatment-seeking obese adults report mean subscale scores with ranges as follows: social/interpersonal functioning, 16.3 to 23.2; self-esteem, 20.0 to 28.2; and activities of daily living, 11.9 to 18.3 [13,32,33]. In our adapted version of the IWQOL-A, no total score is used. Modified from the adult IWQOL, the IWQOL-A differs only in that questions less applicable to adolescents were omitted and terminology was adapted for adolescent use. Questions considered not applicable to adolescents including the Sexual Life subscale (e.g., “I have difficulty with sexual performance”) and questions pertaining to specific aspects of physical health (e.g., “My vision becomes blurred off and on during the day”) were omitted. Questions pertaining to work (e.g., “My career is suffering”), were modified to reflect schoolwork, instead (e.g., “My schoolwork is suffering”). The IWQOL-A has demonstrated good construct validity and test-retest reliability for all subscales other than the comfort with food scale [4], which was therefore excluded. 

The *Children’s Depression Inventory *is a psychometrically sound 27-item self-report questionnaire measuring symptoms of depression in youth [34]. The total score reflects a sum of depressive symptoms related to negative mood, interpersonal problems, ineffectiveness, anhedonia and negative self-esteem. Higher scores indicate greater depressive symptoms.

Body composition was assessed with air-displacement plethysmography (Life Measurement Inc., Concord, CA). Participants were studied after an overnight fast while wearing only underclothes [35].

### 2.3. Statistical Analyses

Analyses were performed with SPSS 16.0. All variables approximated a normal distribution as indicated by skewness < 3 and kurtosis < |10|. A series of analyses of covariance (ANCOVAs) were conducted to determine whether binge eating status (presence *versus* absence) and sex (female *versus* male; independent variables) were associated with poorer QOL in each of the six QOL domains including activities of daily living, health, mobility, self-esteem, social/interpersonal functioning, and work/school (dependent variables). The interaction between binge eating status and sex was also included in each model. Covariates in all ANCOVA models were age (years), race (African American *versus* White, non-Hispanic) and body composition indices including fat mass (%), fat-free mass (kg) and height (cm). Analyses were repeated to account for number of obesity related medical comorbidies (1 *versus* ≥2). This covariate did not contribute significantly to any model and was therefore removed from all analyses. 

Two separate sets of secondary analyses were explored. First, secondary analyses substituted binge eating (presence *versus* absence) in ANCOVA models with LOC eating (presence: objective binge episodes or subjective binge episodes, *versus* absence: objective overeating without LOC or no overeating). Second, since QOL has been associated with symptoms of depression among obese adults with binge eating disorder [36], a series of follow-up ANCOVAs were performed to determine the association of binge eating and sex with QOL after accounting for symptoms of depression. 

In all analyses, differences were considered significant when *p* values were ≤0.05. All tests were two-tailed.

## 3. Results

### 3.1. Participant Characteristics

One-hundred-fifty-eight adolescents were studied. Thirty-five (22.2%) reported at least one binge eating episode in the month prior to assessment (mean ± SD: 3.4 ± 3.9 episodes, range: 1–19). Six participants (3.8%) met DSM-IV-TR criteria for binge eating disorder [1], only one of whom was male. Nine youth met CDI screening cutoff score (19 or higher) for clinical depression. Adolescents with binge eating disorder did not differ from those with binge eating episodes in the absence of the full syndrome on any key variable (all *p*s > 0.17). Therefore, they were grouped together for data analysis. Youth with and without binge eating did not differ with regard to age, sex, race, percent adiposity, or fat-free mass (Table 1). 

**Table 1:** Demographic, Anthropometric, and Psychological Variables of Study Sample by Binge Eating Status.

Variable	Binge Eating (*n* = 35)	No Binge Eating(*n* = 123)	*F*
Sex (% Female)	60	71	1.15
Race (% African-American)	54	60	0.62
Age (years) ^a^	14.8 ± 1.6	14.4 ± 1.3	−1.33
BMI (kg/m^2^)	42.1 ± 9.7	40.9 ± 8.3	−0.71
Percent fat (%) ^a^	48.5 ± 6.0	48.0 ± 6.2	−0.43
Fat-free mass (kg) ^a^	58.0 ± 11.2	55.9 ± 9.4	−1.10
Depressive symptoms ^b^	10.1 ± 6.7	6.2 ± 5.2	−3.22 *
*QOL Domains *^c,d^			
Health	25.8 ± 1.1	23.1 ± 0.6	4.90 *
Social/Interpersonal Functioning	21.8 ± 1.2	19.1 ± 0.7	3.73
Work/School	14.6 ± 0.9	13.9 ± 0.5	0.65
Mobility	18.2 ± 1.0	15.9 ± 0.6	4.07 *
Self-esteem	24.3 ± 1.4	20.2 ± 0.8	6.93 **
Activities of Daily Living	14.9 ± 0.8	13.9 ± 0.5	1.15

* *p* < 0.05; ** *p* < 0.01; ^a^ Mean ± SD; ^b^ Depressive symptoms were measured using the Children’s Depression Inventory [34]; ^c^ Quality of Life (QOL) domains were measured using the Impact of Weight on Quality of Life-Adolescent Version (IWQOL-A) [4,13]; ^d^ Adjusted values ± SEM from ANCOVA models including age, race, percent adiposity, fat-free mass, sex, binge eating, and the interaction between sex and binge eating; marginal means for the main effect of binge eating are reported.

### 3.2. Binge Eating, Sex, and Weight-Related QOL

There was a main effect of binge eating on QOL such that after accounting for age, race, body composition, and sex, youth who reported binge eating had poorer QOL in the following domains: health, mobility, and self-esteem (Table 1; all *p*s < 0.05). There were no significant differences between adolescents with and without binge eating in the QOL domains of activities of daily living (*p *= 0.10), social/interpersonal functioning (*p *= 0.06) or work/school (*p *= 0.43). With regard to sex differences, accounting for binge eating status, race, and body composition, girls reported poorer QOL than boys in the domains of activities of daily living, mobility, self-esteem, and social/interpersonal functioning (*p*s < 0.05), but not health (*p *= 0.12) or work/school (*p *= 0.58). 

**Figure 1:**
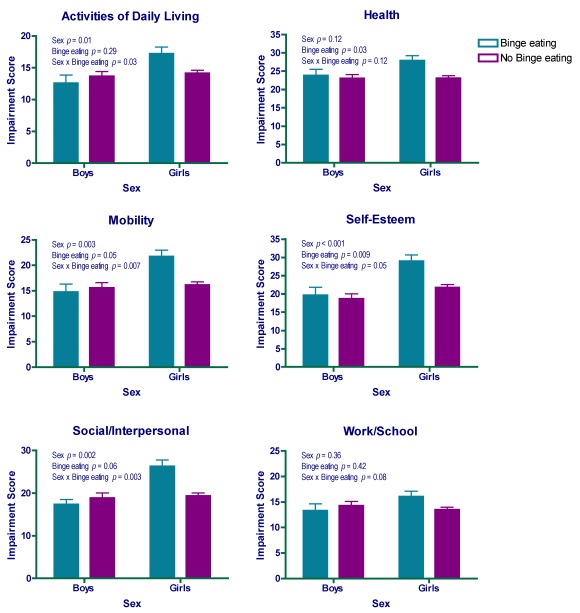
Adjusted means and standard errors for girls and boys with and without binge eating on the quality of life (QOL) domains of activities of daily living, health, mobility, self-esteem, social/interpersonal, and work/school, adjusted for age (years), race (Caucasian *versus* African American), percent body adiposity (%), fat-free mass (kg), and height (cm).

The main effects observed for binge eating and sex were qualified by significant binge eating status by sex interactions for models predicting activities of daily living, mobility, self-esteem, and social/interpersonal functioning (*p*s < 0.05). Examination of these interactions revealed that girls with binge eating reported the poorest QOL compared to all other groups: girls without binge eating, and boys with and without binge eating (Figure 1). Parallel patterns were observed in the QOL domains of health (*p *= 0.12) and work/school (*p *= 0.08), but the binge eating by sex interactions did not reach significance (Figure 1). Results remained unchanged when controlling for number of obesity-related medical comorbidies (1 *versus* ≥2). 

### 3.3. Secondary Analyses of Loss of Control (LOC) Eating, Sex, and Weight-Related QOL

Forty-seven (29.7%) adolescents reported LOC eating (subjective and objective binge episodes), 27 (57.4%) of whom reported objective binge episodes only, 12 (25.5%) of whom reported subjective binge episodes only, and 8 (17.0%) of whom reported a combination of objective and subjective binge episode types. When LOC (rather than binge) eating status was considered in models predicting QOL, the pattern of findings was similar. There were main effects of LOC on health, mobility, and self-esteem domains (*p*s < 0.05) and a non-significant trend on social/interpersonal functioning (*p* = 0.10). Main effects of sex persisted when examining LOC episodes for most QOL domains, including activities of daily living, mobility, self-esteem, and social/interpersonal functioning: girls reported significantly poorer functioning than boys (*p*s < 0.05), but there were non-significant sex differences for the health (*p *= 0.07) and work/school (*p *= 0.30) scales. LOC eating by sex interactions were significant for the domains of mobility, self-esteem, social/interpersonal functioning, and work/school (*p*s < 0.05), such that girls with LOC episodes reported the poorest QOL compared to all other groups. LOC eating by sex interactions were not statistically significant for activities of daily living (*p* = 0.16) or health (*p *= 0.08). 

### 3.4. Secondary Analyses Accounting for Depressive Symptoms

Binge eating and symptoms of depression were correlated (*r *= 0.29, *p* < 0.001). Including age, race, percent adiposity, fat-free mass, height, binge eating status, and sex in models predicting QOL, depressive symptoms were significantly associated with all six health-related QOL domains (*p*s < 0.01). With the addition of depressive symptoms to the models, binge eating was not uniquely related to QOL in any domain. In contrast, the effect of sex on QOL persisted in the domains of activities of daily living, mobility, self-esteem, and social/interpersonal functioning (*p*s < 0.05), though not in domains of health (*p* = 0.26) or work/school (*p* = 0.99). Even after accounting for depressive symptoms, the interactions between binge eating status and sex remained significant in models predicting mobility (*p* = 0.03) and social/interpersonal functioning (*p *= 0.02), such that girls with binge eating reported the poorest QOL compared to girls without binge eating and boys with and without binge eating. Results remained unchanged when controlling for number of obesity-related medical comorbidities. Finally, to isolate a potential unique contribution of binge eating, saved residuals from a regression of binge eating on depression were added to a model using depression and other covariates to predict various QOL domains. Saved residuals from such a model represent the isolated contribution of binge eating (parsing out the contribution of depression). In this set of models, residuals did not add predictive value above and beyond the contribution of depression (*p*s > 0.05).

## 4. Discussion

This preliminary investigation of the associations among binge eating, sex and weight-related quality of life (QOL) in obese, treatment-seeking adolescents revealed that adolescents describing at least one recent episode of binge eating reported significantly poorer QOL in several important domains of functioning: health, mobility, and self-esteem. Across all domains, with the exception of work/school, girls reported poorer QOL than boys. However, in many domains, it was girls with binge eating who reported the poorest QOL compared to all other groups. Said differently, binge eating among girls, but generally not among boys, was related to significantly greater impairment in weight-related QOL in the important QOL domains of activities of daily living, mobility, self-esteem, and social/interpersonal functioning. 

When depressive symptoms were accounted for, binge eating was no longer significantly related to QOL, suggesting that the main effect of binge eating on QOL across boys and girls may be driven primarily via depression. In contrast, girls tended to report poorer QOL than boys across most domains (activities of daily living, mobility, self-esteem, and social/interpersonal functioning) even when controlling for depression, suggesting that the impact of sex on QOL may be independent of depressive symptoms. Analyzing the interaction of binge eating and sex on QOL, girls with binge eating continued to reported worse social/interpersonal functioning and mobility than all other weight-loss treatment seeking adolescents in our sample, independent of depressive symptoms.

Findings from the current study are consistent with several lines of previous research. The present study’s finding that binge eating is associated with poorer QOL in obese adolescents is consistent with literature indicating a relationship between binge eating and QOL among adults [8] and youth with type 2 diabetes [11]. Notably, the present relationship between binge eating and QOL remained significant after accounting for adolescents’ extent of obesity (*i.e.*, percent adiposity), suggesting that the poorer QOL reported by those with binge eating cannot be attributed solely to a greater degree of obesity. Similarly, the finding that girls in the current study reported significantly worse QOL than boys in nearly all domains of functioning is consistent with previous data that obese adolescent females endorse poorer QOL compared to obese males [37,38].

These observations are important to consider in light of apparent interactions between binge eating and sex. The associations between binge eating and QOL were stronger among girls compared to boys in most domains, including mobility, self-esteem, social/interpersonal functioning and work/school. One possible explanation is that obese adolescent girls may experience binge eating as more distressing than boys [39]. Although obese boys certainly are not immune from the social stigma associated with obesity, there is evidence for even greater socio-cultural pressure on adolescent girls to be *ultra* thin [40]. Compared to males, adolescent [41] and adult [42] females tend to experience greater relevance of body weight and shape for identity and self-esteem. Indeed, adolescent girls often report that changing their eating patterns is a means to feeling better about themselves [43]. Consistent with clinical data that females experience binge eating as guilt-inducing and embarrassing [30], it is possible that binge eating impacts girls’ evaluation of themselves across domains (e.g., self-esteem, interpersonal relationships) to a greater extent than their male counterparts. Alternatively, it is possible that problems in weight-related QOL domains, such as difficulties in interpersonal functioning, might promote binge eating behaviors [44].

Similar to several adult studies [36,45,46], we found that depressive symptoms were strongly related to impaired QOL independent of binge eating. However, this finding should be interpreted in light of the significant collinearity between binge eating and depressive symptoms [4]. In the present study, correlation analyses of the association between QOL and depressive symptoms confirmed the expected significant relationship between binge eating and symptoms of depression. Given that obese youth suffer from greater depressive symptoms [47] and report lower psychosocial QOL than non-overweight youth [48], it is possible that the association between depressive symptoms and QOL is even more potent among obese youth than among non-overweight youth. In analyses accounting for depressive symptoms, binge eating was only significantly related to adolescent girls’ weight-related QOL in social/interpersonal functioning and mobility domains. Since most effective interventions for binge eating have been developed from therapies designed to treat depression [44], interventions targeting both mood and eating behavior may simultaneously improve QOL [49]. Nevertheless, the unique relationship of adolescent girls’ binge eating with QOL in some domains (*i.e.*, mobility and social/interpersonal functioning), even after accounting for symptoms of depression, suggests that binge eating may influence QOL by mechanisms other than depressed mood. In other words, QOL may be impacted by factors such as the inconvenience, time, or cost to other pleasurable activities rendered by binge eating. 

Strengths of this study include the use of a structured interview for the assessment of binge eating in a relatively large sample of obese adolescents of varied sex and race/ethnicity. Although there were more girls than boys in the sample, significant sex differences were still detectable. Our findings may be limited in terms of their generalizability to obese, treatment-seeking youth. However, it is highly relevant to understand binge eating and QOL among such adolescents. Investigations of the psychosocial correlates of weight-related QOL are important for the identification of targets upon which to design successful weight-loss programs that can fully address the range of problems and concerns salient for obese treatment-seeking adolescents. Yet, it is important to point out that the associations observed in the current study are cross-sectional, and therefore, conclusions about directionality cannot be made. It is possible that binge eating may promote poorer QOL, or that poorer QOL in domains such as self-esteem may influence binge eating patterns. 

## 5. Implications

Our findings suggest that binge eating may be especially impairing among obese, treatment-seeking adolescent girls in key domains of QOL—social functioning and mobility—but perhaps less so among their male counterparts. Implications for interventions include specific focus on the development of positive interpersonal relationships as well as fostering adaptive attitudes and cognitions related to social functioning. With regard to QOL mobility, practitioners should be mindful of the impact of obesity on the adolescent’s ability to move around in their environment. Interventions might focus on helping the adolescent develop strategies for enhancing mobility delivered in a sensitive manner. Future research should involve an examination of the impact of psychological variables, including binge eating, QOL, and symptoms of depression, on weight-loss treatment outcome. Given preliminary evidence suggesting that girls with binge eating may be differentially impaired in a variety of capacities, elucidation of the role of psychosocial factors in weight-management may enable interventions to be tailored to meet the unique needs of this population. 
